# Mutation analysis of three genes encoding novel LKB1-interacting proteins, BRG1, STRAD*α*, and MO25*α*, in Peutz–Jeghers syndrome

**DOI:** 10.1038/sj.bjc.6602454

**Published:** 2005-03-08

**Authors:** P Alhopuro, P Katajisto, R Lehtonen, S K Ylisaukko-oja, L Näätsaari, A Karhu, A M Westerman, J H P Wilson, F W M de Rooij, T Vogel, G Moeslein, I P Tomlinson, L A Aaltonen, T P Mäkelä, V Launonen

**Affiliations:** 1 Department of Medical Genetics, Biomedicum Helsinki (Haartmaninkatu 8), University of Helsinki, Helsinki FIN-00014, Finland; 2 Molecular Cancer Biology Program, Institute of Biomedicine and Helsinki University Central Hospital, Biomedicum Helsinki, Helsinki, Finland; 3 Laboratory of Vascular and Metabolic Diseases, Department of Internal Medicine, Erasmus Medical Center, Rotterdam, The Netherlands; 4 Unfallchirurgie, Universitätsklinikum, Heinrich Heine Universität, Düsseldorf, Germany; 5 Allgemein- und Viszeralchirurgie, Universitätsklinikum, Heinrich Heine Universität, Düsseldorf, Germany; 6 Molecular and Population Genetics Laboratory, Imperial Cancer Research Fund, 44, Lincoln's Inn Fields, London WC2A 3PX, UK

**Keywords:** Peutz–Jeghers syndrome, *BRG1*, *STRADα*, *MO25α*, *LKB1*

## Abstract

Mutations in *LKB1* lead to Peutz–Jeghers syndrome (PJS). However, only a subset of PJS patients harbours *LKB1* mutations. We performed a mutation analysis of three genes encoding novel LKB1-interacting proteins, *BRG1*, *STRADα*, and *MO25α*, in 28 *LKB1*-negative PJS patients. No disease-causing mutations were detected in the studied genes in PJS patients from different European populations.

Peutz–Jeghers syndrome (PJS) is an autosomal dominantly inherited disorder characterised by mucocutaneous pigmentation, hamartomatous polyps of the gastrointestinal tract, and a predisposition to cancer at a young age (Giardiello *et al*, 1987, 2000; Hemminki, 1999). The majority of cancers in PJS patients occur in the stomach and upper small bowel, whereas the most frequently diagnosed extraintestinal cancers are those of the pancreas, breast, and testis (Giardiello *et al*, 1987).

Inactivating mutations in the serine–threonine kinase *LKB1* (*STK11*) have been causally linked to PJS (Hemminki *et al*, 1997, 1998; Jenne *et al*, 1998). However, 30–82% of PJS patients have no detectable *LKB1* mutations, suggesting locus heterogeneity (Lim *et al*, 2003, and references therein). Moreover, some families have been reported to be clearly unlinked to *LKB1* locus (Mehenni *et al*, 1997; Olschwang *et al*, 1998). Linkage analysis has previously yielded a second locus on chromosome 19q13.4, and recently, a translocation breakpoint was mapped to the same locus, but several genes within this region have subsequently been excluded from having a role in PJS (Mehenni *et al*, 1997; Buchet-Poyau *et al*, 2002; Hearle *et al*, 2004). Thus, current candidates for the second PJS loci are genes known or suggested to interact with *LKB1* either by direct association or functionally.

LKB1 has been suggested to mediate its cellular functions through interactions with a number of proteins including LIP1, BRG1, MO25*α* and STRAD*α* (Marignani *et al*, 2001; Buchet-Poyau *et al*, 2002; Baas *et al*, 2003; Boudeau *et al*, 2003). Of these, BRG1 is appealing, as Brg1 heterozygous mice form epithelial subcutaneous tumours (Bultman *et al*, 2000). LKB1 has been proposed to associate with and stimulate BRG1, and LKB1 might have a role in BRG1-dependent growth arrest (Marignani *et al*, 2001). Both STRAD*α* and MO25*α* have been identified by two independent methods: by probing AMPKK fractions using antibodies against LKB1, STRAD*α*, and MO25*α*, and by purification of LKB1 binding proteins (Baas *et al*, 2003; Boudeau *et al*, 2003; Brajenovic *et al*, 2004). STRAD*α* is the first identified LKB1 substrate *in vivo*. Furthermore, both STRAD*α* and MO25*α* have been shown to translocate LKB1 from the nucleus to cytoplasm, and interestingly, previous studies have shown that cytoplasmic localisation is significant for LKB1 growth suppressive function (Tiainen *et al*, 1999, 2002).

Of the LKB1-interacting proteins, only LIP1 (Buchet-Poyau *et al*, 2002) has been analysed as a candidate for a second PJS locus. Here, we have extended this approach by analysing three interesting genes encoding LKB1-interacting proteins: *BRG1*, *STRADα*, and *MO25α.*

## MATERIALS AND METHODS

A total of 27 *LKB1* mutation-negative PJS patients from four different European countries (seven from Finland, eight from UK, two from the Netherlands, and 10 from Germany) were included in the study. A diagnosis of PJS was made when the patient presented with two or more PJS polyps, one polyp and typical pigmented lesions, or one polyp and a family history of PJS (Tomlinson and Houlston, 1997). Additionally, one Finnish patient who had been diagnosed with a single PJS polyp at the age of 29 years, with unknown family history, was included in the mutation screening. Sporadic disease was diagnosed in 15 (54%) patients and familial disease in 10 patients (36%). No family history was available from three patients. DNA extraction was performed using standard methods. Healthy controls were obtained from the Finnish Red Cross Blood Transfusion Service.

All 34 coding exons and the flanking intronic sequences of *BRG1* (NM_003072.1) were amplified in 38 fragments. Denaturing high-performance liquid chromatography (DHPLC) method was used in preliminary mutation screening with automated HPLC instrumentation (Agilent Technologies, Palo Alto, CA, USA), and Helix DNA 50 × 3.0 mm × 1/4″ analytical column (Varian Inc. Palo Alto, CA, USA). Optimal melting temperatures for each amplicon were obtained using an algorithm at the Stanford DHPLC Melt program web page (http://insertion.stanford.edu/ melt1.html). The analytical acetonitrile gradient was composed by mixing Helix BufferPak A for DHPLC and 55–75% B (Varian) at flow rate of 0.4 ml min^−1^. All samples displaying aberrant or ambiguous pattern in DHPLC were reanalysed by sequencing with ABI 3730 sequencer (Applied Biosystems, Foster City, CA, USA). Polymerase chain reaction (PCR) primers are available from the authors upon request.

The mutation screening of the 12 coding exons of *STRADα* (BK001542.1), the eight coding exons of *MO25α* (NM_016289.2), and the flanking intronic regions was performed by genomic sequencing using Applied Biosystems 3730 sequencing analyzer. Polymerase chain reaction conditions and primer sequences are available from the authors upon request.

The potential effect of silent polymorphisms, intronic, and UTR changes to splicing were predicted by computational methods using NetGene2 splice site prediction web server (http://genome.cbs.dtu.dk/servi ces/NetGene2/). Silent polymorphisms in exons 5 and 27 of *BRG1* were predicted to alter splicing *in silico*. The polymorphism in exon 5 was predicted to increase the probability of cryptic acceptor splice sites flanking the mutation site, whereas in exon 27, a new cryptic donor splice site was predicted. To study the effect at the RNA level, RNA from cultured lymphoblasts was extracted, and reverse transcriptase–PCR (RT–PCR) reactions and cDNA amplifications were performed using standard protocols. Potential splicing variants were visualised by 2% agarose gel electrophoresis. Furthermore, to detect possible effects of altered splicing to expression, real-time PCR was performed using ABI Prism 5700 sequence detection system (Applied Biosystems) and SYBR Green PCR master mix (Applied Biosystems). cDNA samples from two unaffected individuals were used as controls. Primers used were as follows: 5′-AAGCCCTGGCCTGAAGGA-3′ and 5′-TGCGGGGGAATCAGCTTCT-3′ for the exon 5 change and 5′-GGCGTCAACCCCGACTT-3′ and 5′-TGCGCATGAACAGAT CAAACTC-3′ for the exon 27 change. The reverse primers were designed so that they located in the potentially spliced cDNA segment. *GAPDH* and *TBP* were used as control genes to normalise the reactions.

## RESULTS AND DISCUSSION

**Figure 1:**
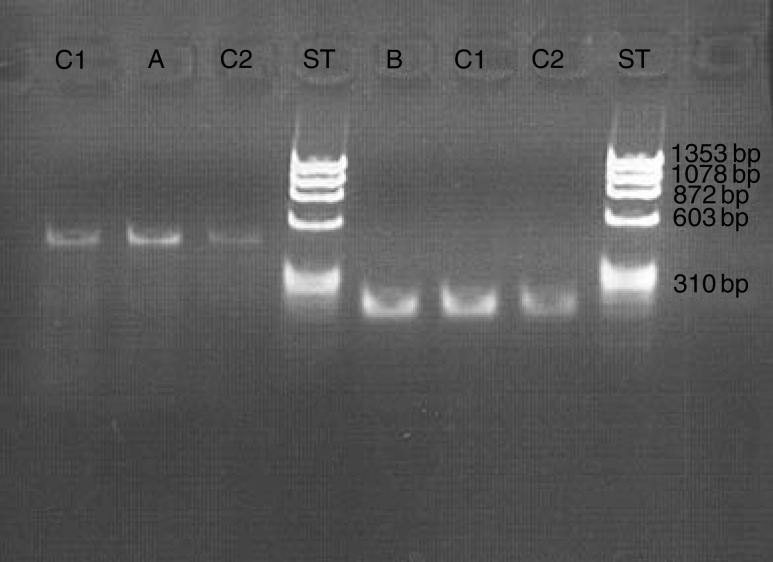
Effects of the novel *BRG1* silent polymorphisms to splicing were determined by RT–PCR. No difference in amplicon size was observed between mutated samples (A=Pro327Pro, 478 bp and B=Pro1297Pro, 296 bp) and controls (C1 and C2). ST=size standard (DNA marker).

To determine whether *BRG1*, *STRADα*, or *MO25α* are mutated in *LKB1* mutation-negative PJS patients, we performed a mutation screening in a set of 28 patient samples. Mutation analysis of *BRG1* was performed by DHPLC. The sensitivity of the method has been shown to vary between 93 and 100%, consistently exceeding 96% (Xiao and Oefner, 2001). In *BRG1*, two novel silent changes were observed (Table 1). A Pro327Pro (c.980A>C) change in exon 5 and a Pro1297Pro (c.3890C>G) change in exon 27 were both found in 4% (one of 28) of the subjects (allele frequency 2%). The Pro1297Pro variant was also detected in healthy controls (two of 188, allele frequency 0.5%), whereas DHPLC analysis of 267 healthy controls revealed no Pro327Pro changes. Splicing effects of these variants were subsequently tested *in silico*, and the Pro327Pro change was predicted to alter acceptor splice site of exon 5 and the Pro1297Pro change to create a potentially new donor splicing site. To further discern the effects of these variants to splicing, RT–PCR analysis was performed. However, no alternate transcripts were detected at the RNA level (Figure 1). Furthermore, quantitative real-time PCR was performed to detect possible changes in expression caused by alternative splicing. Means of normalised expressions of *BRG1* were 1.5 (s.d. 0.14) and 1.4 (s.d. 0.31) in patient with Pro327Pro and normal controls, respectively. Means of normalised expressions of *BRG1* were 1.6 (s.d. 0.28) and 1.4 (s.d. 0.26) in patients with Pro1297Pro and normal controls, respectively. Thus, no decrease in *BRG1* expression levels was observed in patients with Pro327Pro and Pro1297Pro mutations when compared to controls.

Five previously reported silent polymorphisms were detected: Ala503Ala, His508His, Asp1351Asp, Asp1528Asp, and Asp1628Asp (Valdman *et al*, 2003; Medina *et al*, 2004, http://www.ensembl.org). Also, a previously described missense polymorphism, Tyr372His, was found in two PJS patients (Medina *et al*, 2004). Additionally, six intronic variants were observed (Table 1). Splicing effect of all intronic variants was subsequently tested *in silico*. One of the variants, IVS17-4C>A, gave little evidence of alternative splicing. The IVS17-4C>A change was found in one PJS patient, but not in 186 healthy controls (Table 1). The effects of this variant could not be studied at the RNA level, because no sample was available.

In *STRADα*, a single-nucleotide polymorphism, a T>C change at position 96 (allele frequency 63%), was observed in the noncoding sequence of exon 2. Moreover, three intronic polymorphisms were detected. Sequencing of *MO25α* revealed one intronic and two 3′UTR polymorphisms. The detected variants in *STRADα* and *MO25α* (Table 2) were not predicted to affect splicing *in silico*.

Several previous studies support the hypothesis of locus heterogeneity of PJS. However, linkage data and candidate gene approach have not yielded a second PJS gene (Buchet-Poyau *et al*, 2002). Little has been known about LKB1-interacting proteins until recently. Cellular functions suggested for LKB1 to date include an ability to induce cell cycle arrest through p21, involvement in p53-dependent apoptosis pathway and VEGF signalling, involvement in BRG1-dependent chromatin remodelling, induction of colon epithelial cell polarity, and contribution to cell stress censoring through activation of the AMPK kinases (Tiainen *et al*, 1999; Karuman *et al*, 2001; Marignani *et al*, 2001; Ylikorkala *et al*, 2001; Baas *et al*, 2003; Hawley *et al*, 2003). Furthermore, the recent demonstration that LKB1 acts as an *in vivo* activating kinase for at least 13 other kinases suggests that LKB1 will be involved in several as yet uncharacterised functions (Lizcano *et al*, 2004).

Proteins interacting with LKB1 and enhancing its tumour suppressor function are attractive candidates for the second PJS locus. Here, we analysed three genes encoding LKB1-interacting proteins: *BRG1*, *STRADα*, and *MO25α*. While our approach is unable to detect some mutation types, such as large deletions, the results suggest that germline mutations in these three genes have no major contribution to PJS. These genes were selected as the best candidates at the initiation of the study. Nevertheless, the recent identification of a number of *in vivo* substrates of LKB1 as well as the identification of MO25*β* and STRAD*β* provide several novel interesting candidates to be analysed in subsequent studies (Boudeau *et al*, 2003; Lizcano *et al*, 2004).

## Figures and Tables

**Table 1:** Summary of *BRG1* polymorphisms identified in the study

**Location**	**Variant**	**Allele frequency (%) in PJS (no. of patients)**	**Allele frequency (%) in controls**	**Reference**
Exon 5	Pro327Pro, c.980A>C	2 (1/28)	0 (0/267)	This study
Exon 5	Tyr372His, c.1114T>C	4 (2/28)	—	Medina *et al* (2004)
Exon 8	Ala503Ala, c.1509A>C	2 (1/27)	—	Medina *et al* (2004)
Exon 8	His508His, c.1524T>C	19 (8/27)	—	Ensembl^a^
Exon 27	Pro1297Pro, c.3890C>G	2 (1/28)	0.5 (2/188)	This study
Exon 28	Asp1351Asp, c.4333C>T	7 (3/28)	—	Valdman *et al* (2003)
Exon 31	Asp1528Asp, c.4584C>T	4 (2/28)	—	Ensembl^a^
Exon 33	Asp1628Asp, c.4886T>C	9 (4/28)	—	Ensembl^a^
Intron 9	IVS9+29G>A	2 (1/25)	—	This study
Intron 9	IVS9−37T>C	32 (13/24)	—	This study
Intron 13	IVS13+12C>T	2 (1/28)	—	This study
Intron 17	IVS17+55G>A	5 (3/28)	—	This study
Intron 17	IVS17−4C>A	2 (1/28)	0 (0/186)	This study
Intron 32	IVS32−44C>T	2 (1/28)	—	This study

—, not tested in this study.

a http://www.ensembl.org.

**Table 2:** Summary of *STRADα* and *MO25α* germline variants and allele frequencies detected in PJS patients

**Gene**	**Location**	**Variant**	**Allele frequency (%) (no. of patients with mutation)**
*STRADα*	Exon 2	nt.96T>C	63 (25)
	Intron 2	IVS2+15A>G	2 (1)
	Intron 5	IVS5−30G>C	29 (14)
	Intron 10	IVS10+87	64 (25)
			
*MO25α*	Intron 5	IVS5+130T>A	2 (1)
	3′UTR	nt.1419_1420delTT	12 (7)
	3′UTR	nt.1552G>C	5 (3)
